# Hospital and Institutional News

**Published:** 1912-12-14

**Authors:** 


### HOSPITAL AND INSTITUTIONAL NEWS.

### OUR CHRISTMAS APPEAL NUMBER.

This week, as previously announced, The Hospital is supplemented by a specially illustrated Christmas Appeal Number, which sets forth the needs of the hospitals at the present time. Besides two special articles, entitled respectively "How our Hospitals Keep the Feast," and "What Christmas means to Institutional Workers," illustrations are given showing the difference between Christmas inside and outside the hospital. We hope that our readers will make every effort to distribute the Appeal Number, which is to be obtained separately for the modest sum of one penny, as widely as possible. It is intended for all within reach of hospital committees, who may be brought to realise what the voluntary system means by seeing it in the typical aspect of Christmas cheerfulness, and who may turn from this to the summarised needs of the various institutions, and give their support to those which most appeal to them. All classes of hospitals are represented, but the inspiring spirit is the same, and its happiest expression is seen perhaps in the freely-given service of countless workers at Christmas time.

### THE PROGRESSIVE DEMAND FOR MENTAL RESEARCH.

The possibility of obtaining State aid for scientific research in mental diseases was considered at a conference of mental hospital representatives at the Guildhall last week, which is fully reported elsewhere in this issue. The need for such research is, however, so real and so neglected that we make no apology for summarising the chief speeches in this column. The Chairman, Mr. Morgan Thomas, Lord Mayor of Cardiff, the Corporation of which had summoned the conference, asked why mental diseases should be deprived of their grants for research work which the Local Government Board often gave in other directions. Sir George Savage then moved a resolution in favour of grants being given, and urged that they should be made through the medium of and upon the recommendation of the Lunacy Commissioners. Isolated efforts were not sufficient for the thorough investigation of mental disease. Dr. J. Shaw Bolton, superintendent of the West Riding Mental Hospital, remarked that 135,000 persons were certified as suffering from mental disease, and that this number was increasing. Any attempt to cure insanity, he insisted, could be made only as a result of research which should be carried out in all parts of the country. This work which was for the benefit of the public, the public should pay for and the Government should help. Dr. James Soutar, President of the Medical Psychological Association, advocated the establishment of clinics for the diagnosis and treatment of cases of incipient disorder. This proposal, which was considered in The Hospital only recently, was warmly supported by Sir Norval Helme, M.P., who represented the Lancashire Asylums Board, but he contended that the grants should not be made by the Lunacy Commissioners, who were not answerable to Parliament. Dr. T. W. McDowall said that the great difficulty about the clinic was that the patients themselves would not recognise the early stages of insanity. So much for the discussion.

### HOW THE MENTAL HOSPITALS ARE STARVED.

But the interest of the Conference does not end with these typical expressions of opinion. That differences exist is largely due to the absence of the correction which research and its teachings alone can give. We are glad to note, therefore, that a deputation is to be appointed to wait upon the Lunacy Commissioners and the Home Secretary. The moment is symptomatic if not opportune. The isolation hospital for infectious disease is now seen to be insufficient, and the result is that the County of London is to have a specially appointed bacteriologist of its own to undertake research work. This, we may be sure, is only the beginning. It is time that the criticisms which have long been made against mental hospitals as being rather houses of detention than curative institutions should crystallise into definite constructive proposals. Statistics on the subject are known to be based on such an absence of definition (if a ridiculous phrase may be used to express a ridiculous truth) that no mental authority regards them as other than misleading. The mental hospital is the shining example of the publicly controlled institution. It is unthinkable, therefore, that it should continue to be starved of that relatively small expense which alone can make its activities preventive.

### THE DIFFICULTIES OF THE POOR-LAW MEDICAL OFFICER.

The difficulties attending the lot of a medical superintendent of an infirmary, especially when the institution is not of an up-to-date character, are borne out by a report submitted to the Lambeth Board by Dr. A. Lionel Baly, the medical officer. After pointing out that 12 per cent. of the patients were on the danger list, thus greatly increasing the duties of the extremely hard-worked staff, he reminds the Board that he has frequently requested improvements in the building and appliances to minimise the work of the staff. His application for new flushing apparatus in the ward lavatories has been completed, it is true, in nine wards; but there are still nine to be done. The antiquated beds with wooden frames, that require a great amount of labour to keep clean, have recently been supplemented by fifty new beds, which were bought to replace some that were actually unsafe. The old-fashioned lifts, which entail a considerable amount of labour besides unnecessary waste of time, are to be replaced, Dr. Baly understands, by a type that can be safely used by any member of the staff. The telephone, he remarks, is broken down completely, whilst the bad condition of the ward floors is very fatiguing to those constantly standing or walking upon them. In face of these facts the Board has concurred in his views that up-to-date appliances must be adopted. A further 100 new beds are to be purchased at once. Dr. Baly is to be congratulated on his insistence, for, as Dr. C. T. Parsons recently reminded our readers, the importunate widow is the type to be followed by the Poor-Law medical officer when faced by difficulties of this kind.

### A NEW FORM OF CHRISTMAS ENTERTAINMENT.

Now that cinematographs are to be domesticated, so to speak, and that an enterprising firm has presented to several hospitals in London their new apparatus for giving moving pictures in private houses, we hope that one or other of the institutions will send us their experience of its advantages or drawbacks. Since opinions may vary, we hope to receive several descriptions. A new form of Christmas entertainment is bound to interest hospitals generally, and, if one has been found, which to its obvious virtue of quietness combines a complicated result without exhausting preparation, the firm devising it is entitled to our thanks. Any risk of fire is claimed to be satisfactorily safeguarded, but since the *Titanic* disaster gave such a very unpleasant flavour to the adjective unsinkable, as applied to passenger vessels, fireproof and similar adjectives are bound to suffer by analogy in public esteem. We shall be interested to hear the result of this new departure, and to learn whether the patients proved enthusiastic over the innovation.

### THE BRITISH MEDICAL ASSOCIATION AND THE CHANCELLOR.

Whatever may be the final outcome of the negotiations between the medical profession through its premier organisation and the Chancellor of the Exchequer, it cannot be said that the latter has shown much disposition to avoid a rupture during the past ten days. First of all he propounded to the Association a list of concessions, the practical value of which was almost nothing; and then he allowed—probably encouraged—the Insurance Commission to issue to every practitioner a pamphlet setting forth the conditions of service which reads like an attempt to go behind the British Medical Association altogether. That this pamphlet should have been sent out whilst the members of the Association were considering their votes and their policy is most unfortunate from every point of view. We cannot believe that this premature publication has done or can do anything to further the cause of peace or the success of the Act.

### A DISINGENUOUS OFFICIAL PAMPHLET.

For one thing, the "explanatory statement as to medical benefit," published by authority, with the Royal Arms on the cover, contains misstatements of fact. Thus, in the 23rd paragraph it is stated that under the capitation system of remuneration, the practitioner "can confidently rely" on six shillings and sixpence per insured person on his list, plus sixpence (for tuberculosis) and a possible sixpence from the Drugs Suspense Fund. The facts are, of course, that the practitioner can rely on nothing of the sort. All he can rely on is such residue of these sums as remains for distribution in his district after payments for mileage, surgical operations, night fees, anæsthetics, and other extras have been distributed—possibly to medical men not upon the panel at all. Paragraphs 48 and 50 are so drawn up as to be calculated to mislead the unsuspecting. Insurance Committees must consult the local Medical Committees on certain points, it is emphatically remarked; but no mention is made of the fact that the Insurance Committees need take absolutely no notice of the advice they receive, and that they are mainly composed of representatives of those very Friendly Societies under whose tyranny the profession has been so much ground down.

### THE RECALCITRANT MINORITY'S MANŒUVRE.

We turn from this aspect of the administration of medical benefit to a circular sent out broadcast to doctors last week by some twenty medical men who proclaim their willingness to accept the Chancellor's terms. Evidently some of these men have signed pledges of the British Medical Association; for they declare that such pledges can no longer be binding in the new conditions. After this unpromising beginning, it is not surprising to find that the whole affair is an attempt to wreck the Association. Those who have signed it are for the most part quite unknown in the profession; and the exceptions to this statement are some of those who unsuccessfully tried months ago to get the profession to knuckle under to the Chancellor on the old terms, now amended. Apparently, they feel this difficulty, for they admit that "the cohesion of the Association has had a powerful effect in securing better terms." Yet this cohesion they attempted to destroy months ago, and are now attempting to destroy again. They proceed to some vague talk about a medical pension fund, but no information is vouchsafed as to who is to provide the necessary funds. We cannot wish them success in their campaign of opposition to the British Medical Association, and we trust that no one but themselves will attend their meeting.

### PIONEER WORK AT SPEZIA.

A correspondent writes to us appealing for an English nursing staff to assist the municipality of Spezia in opening the Public Civic Hospital which was built there four years ago, and which an effort is now being made to open. An old convent in the town is apparently being used now as a hospital, but "the nursing arrangements are deplorable, and [the medical staff] cannot organise the new nursing staff properly without English help." The lady who undertakes the work of organisation, the writer remarks, will require experience, business tact, and patience against official stinginess and possible unfairness, adding that "it would be desirable to have very strict agreements binding the municipality for a number of years." For particulars of the appointments for a matron and nurses our readers are referred to the "Institutional Worker" section of this week's issue. Any further information, we understand, can be obtained from the Rev.

H. H. Pullen, Casa Alberto, Spezia. The opportunity for pioneers here presented should not be entered upon until applicants are fully satisfied about the conditions which they will have to expect.

### THE MAUDSLEY MENTAL HOSPITAL.

The London County Council propose to proceed with the erection of the Maudsley Mental Hospital as soon as the plans have been approved by the Home Secretary. The plans which the Asylums Committee have prepared provide for three main buildings—the administrative building in which accommodation for part of the resident staff, an out-patients' department, pathological laboratory, and the general stores would be placed; and two hospital buildings of three floors, having on each floor a complete ward for eighteen patients and two attendants. The total number of patients to be accommodated is therefore 108. The provision of accommodation for the pathological laboratory, which was an essential condition of Dr. Maudsley's original gift of £30,000, will involve the disuse of the present laboratory buildings at Claybury Mental Hospital, for which a new scheme is being considered. The total cost of the buildings, exclusive of equipment, is estimated at £48,000, which taken on the basis of 108 beds is equivalent to £444 8s. 10d. per bed. It is estimated that the equipment of the institution will cost approximately £74 a bed.

### HOW THE HIGH COST PER HEAD IS EXPLAINED.

The Finance Committee of the Council point out that the cost of the buildings and engineering work in an ordinary mental hospital is between £230 and £240 a bed; where, however, a pathological laboratory and an out-patients' department are not provided, and that with the omission of these requirements the cost is reduced to just over £385 a patient. The Maudsley Hospital, moreover, is designed to accommodate acute cases alone, and only a comparatively small number of these. It is urged, therefore, that a fair comparison cannot be made between the cost of a large hospital for two thousand or more patients, many of whom are chronic cases, and the new building. The cost of the equipment, £74 a bed, which is very large compared with the £24 a bed for 2,066 beds in the case of the Council's newest mental hospital, is met by like considerations.

### THE BURDEN OF INSURED PATIENTS.

The house committee of the London Hospital has presented a report on the position of the hospital under the Insurance Act, which has been adopted and can be summarised as follows. The cost of the insurance of the hospital's employees is about £850 a year. Of the patients who have attended since July 15, 45 per cent. were really insurable and 25 per cent. were actually insured. If the hospital continued to treat these insured persons next year it would do so at a cost of more than £20,000 for in-patient treatment and of more than £5,000 for out-patient treatment. The committee, it is interesting to note, are convinced that a great number of these patients must be treated because, although the Act promised "adequate medical relief and treatment," they were not in a position to give it. Serious operations and such special treatments as the $x$-ray or Finsen light, would necessitate institutional treatment for the poor. The position that the Committee proposes to take up is that while not disposed to refuse treatment under certain conditions, they were not at all sure that that treatment ought to be given freely. Slight cases, they agreed, should not be treated but should be referred back to the doctor on the panel. Thus if the London Hospital continues to treat insured patients it will have to spend £25,000 of its total expenditure upon their behalf.

### THE STARTING OF A FESTIVITIES FUND.

There are still many hospitals which have not put their Festivities Fund on a proper basis. We, therefore, remind them again of the secretary who makes use of the early weeks of December by beginning with requests to members of his committee, and ending with the sending out of one hundred or more letters asking for gifts in kind. Here we quote again an actual list: "Turkeys, cheese, fruit, flowers, evergreens, plants, a Christmas tree, tobacco, papers, scrap-books, crackers, sweets, cakes, jellies, loan of silver-plate, earthenware, and cutlery." Experience which has found these a useful list of requisites may afford ideas to hands less practised, who hope to organise a Festivities Fund, but have not yet done so.

### THE ADVANCE OF THE LINEN LEAGUE.

A quarter of a century ago, when The Hospital was still a young newspaper, the Linen League as a recognised organisation had no existence. Indeed, it may be said not to have existed at all. At the close of the year, therefore, when annual presentations of linen are taking place at many of the hospitals of the country, it is interesting to summarise the process of this branch of hospital work. Our advocacy of Linen Leagues was emphatic from the first, and we pointed out with insistent frequency that trial invariably disproved the objections which usually then accompanied a plea for their inauguration. As a matter of fact the Linen League was the precursor of many subsidiary but vitally important movements on which the modern hospital depends for its support. It was the first step in that process of decentralisation which is shown in the workingmen's collection, the Samaritan Fund, the almoner, the Ladies' Association, the League of Mercy—organisations whose functions are twofold—to supply the need for which they were specially created, and to arouse sustained interest in different sections of the public. It is no uncommon thing to-day for a hospital with about 100 beds to receive from £200 to £300 worth of linen from its Ladies' Association or Linen Guild. When it is remembered that the best organised of these make a practice of urging their members to make actual articles rather than to send money for the purchase of them, that others have branches in schools among girls of various classes, it will be seen how wide is the interest that such an organisation can create not merely in its own success but in the institution which it serves. The Linen League as such has not received its due meed of public praise. We therefore write its eulogy to-day with the fatherly interest of one of those who were the first to recognise and foresee its value.

### THE NAMING OR NUMBER OF ASSESSORS.

Architectural papers are discussing whether the name of an assessor should be made known before competitive designs are submitted. The question arose through Mr. C. F. A. Voysey's appeal to the Royal Institute to exert its influence to prevent the naming of assessors before competitors send in their designs. His object was that each architect should design in his best manner without being tempted to play to the style or type of design known, by the assessor's works, to be favoured by him. In our columns of July 20, page 412, we dealt at some length with this question, and suggested that in consequential works not less than two assessors should be appointed. This would satisfy Mr. Voysey's legitimate appeal, and at the same time meet the views of many architects who do not agree with Mr. Voysey's view of the question. It is interesting to note that Mr. W. A. Pite, the successful competitor for the new King's College Hospital, pleads in last week's *Builder* for a jury of three assessors, and is not particular whether they are named or not before designs are submitted.

### A SONNET TO THE LONDON HOSPITAL.

Patients are known to have very diverse ways of expressing their gratitude, but one which has always appeared to us peculiarly odd is the desire of many to say in rhyme the thanks which sound much more sincere in prose. We have just received, for instance, a sonnet to the London Hospital by "an ever grateful patient." While requesting its publication, under no more definite authorship than this, the writer expresses the opinion that the poetical merit of his sonnet is "nil," but that it is the best expression of gratitude possible from him. We beg to differ. The accompanying letter is a far better expression of gratitude than the sonnet. But we think that the London Hospital is entitled to know that another of its ex-patients has been inspired by the kindness meted out to him to try the most difficult poetic form in the English language. No small tribute to the affection which it inspires! Knowing how much efforts like these mean to their authors, we record his appreciation, while being precluded by his own estimate from publishing it. Let him take courage however: "all bad poetry springs from genuine feeling," said one of the two modern English authors who share Shakespeare's popularity in Germany.

### MORE CHANGES AT ST. BARTHOLOMEW'S.

A vacancy for a surgeon is being advertised in connection with St. Bartholomew's Hospital, owing to the retirement of Mr. Bruce Clarke, the senior member of the surgical staff. Mr. Clarke has served Bart.'s long and faithfully, and has always been conspicuous for his interest in the newest discoveries and advances in surgery, as well as in the younger men of his own and other hospitals. The senior assistant surgeon is Mr. Bailey, and there is no reason to suppose that his promotion will be denied to him. The vacancy thereby arising on the junior visiting staff will no doubt arouse keen competition, for St. Bartholomew's is well off at present in surgeons (and physicians) eager to win their spurs; and this, notwithstanding the fact that there has been a good deal of promotion there in the last few years. Three or four years ago Mr. Harrison Cripps, Mr. Lockwood, and Mr. Clarke were all on the staff; and it was not very many years previously that Mr. Howard Marsh left to become Regius Professor at Cambridge, and Sir Henry Butlin retired through press of other work. So that incidental vacancies on the surgical side have been unusually common in recent years at the senior hospital of London.

### THE BONNETT LABORATORY AT CAMBRIDGE.

The new clinical laboratory to be erected at Addenbrooke's Hospital, Cambridge, is the gift of Mrs. Bonnett—not Mrs. Burnett as has been incorrectly stated. The laboratory is Mrs. Bonnett's gift in memory of her son, and it is interesting to recall that Mr. John Bonnett was Mr. Richard J. Coles' predecessor in the office of secretary.

### OVERCROWDING IN THE TUBE LIFTS.

It is time that an end should be put to the excessive overcrowding that is allowed to take place in the lifts conveying passengers at the Tube Stations. This evil is one which is distinctly encouraged by the employees, and takes place not only at the times of the morning and evening rushes, but may be observed at all times during the day. The packing of human beings in such a confined space ought not to be tolerated, and we are glad to record that protests are being made almost daily. No doubt the passengers themselves are partly to blame. There are always to be found persons who will subject themselves and others to discomfort for the sake of saving a few seconds of their time. But it is the duty of the authorities to see that this very unhygienic, not to say dangerous, practice is reduced to a minimum. During the winter months, and especially on damp days, this concentrated aggregation of all sorts and conditions of men and women is not only exceedingly unpleasant, but, in the close atmosphere of the Tube Railways, is highly conducive to the spread of infectious disease, especially scarlet fever. These conditions rival the cheaper and worst-ventilated cinematograph shows in assisting to fill our isolation hospitals.

### THE SCHOOL CHILDREN QUESTION: IMPORTANT DECISION.

In the Court of Session, Edinburgh, the Lord President, Lord Dunedin, gave an important judgment with regard to the powers of Scottish School Boards to provide medical and dental treatment out of the rates. The ratepayers had brought the case in order to have decided what powers the

Glasgow School Board possessed with regard to dental treatment. The Court decided that the Board was entitled to provide medical examination and supervision, but not treatment, even though the parents of the children were unable through poverty or other causes to provide it themselves.

### THE FATE OF THE LEICESTER DISPENSARY.

THE governors of the Leicester and Leicestershire Provident Dispensary have been compelled to issue the following circular to their members: "On behalf of the Board of Governors I beg to give notice that the Dispensary and all the Branches will be closed on December 31, 1912, owing to the fact that all the medical officers have resigned their positions on the dispensary staff. W. Dalrymple, Manager." The dispensary medical officers are between thirty and forty in number, and their intended resignation was announced in June last, when the profession as a whole decided to resign all contract practice. The dispensary, which was founded in 1833 and became a provident dispensary in 1862, has fourteen branches in various parts of the town, and in 1911 had on its books the names of some 60,000 members. To prevent its complete closing the medical staff are stated to be discussing some form of combination with the other local members of the profession, by which it is hoped to rent the central dispensary and four branches which happen to be the freehold property of the Board. In the meantime, a circular is being awaited explaining the situation. The first-fruits of the Insurance Act at Leicester do not seem very refreshing, and, we fear, will not prove to be rare.

### THE TOMB OF A HOSPITAL FOUNDER.

THE burial place of John Harrison, the founder and first surgeon of the London Hospital, has now been located in the churchyard of St. Paul's Church, Deptford. The house committee announced at the recent quarterly meeting that no memorial existed in the church or churchyard to his memory. So by the Rector's consent the house committee has arranged to put up at the personal cost of its members a tablet bearing the subjoined inscription: "In memory of John Harrison, founder and first surgeon of the London Hospital, who died in 1753 and was buried in this churchyard. His body lies here. His work continues at the hospital." It would be hard to improve on this epitaph, which has the merit of brevity, a true merit, by the way, since it is insincerity which usually delights in long inscriptions. The voluntary system has won its laurels and maintained its hold on our affections by the tradition of personal service which it has created. This tradition is fittingly honoured by such a memorial as this, which, it will be noted, is the personal gift of John Harrison's successors in office.

### CLINICAL PATHOLOGY AT MICROSCOPIC FEES.

ONLY recently we had occasion to comment on a very extraordinary project for conducting clinical bacteriology and pathology at microscopic fees, started in conjunction with a firm of patent food manufacturers (THE HOSPITAL, November 30, 1912; p. 237). Since then another new enterprise has been launched, under the ægis of no less a person than Professor A. von Wassermann. The "Institute" bears his name, is stated to be under his constant control, and is to undertake the diagnosis of syphilis by his original method and no other. The announcement comes from an address in Lloyd's Avenue, of all places in London; but no information is given concerning the "medical specialist" who will supervise the work. The fee for testing blood for the Wassermann reaction is fifteen shillings, a sum almost as much below current rates as those of the organisation we remarked upon a fortnight ago. It is not long since the technique of Wassermann's reaction was known only to a handful of pathologists; and eight guineas was, we believe, the tariff at that time. Prices have declined markedly since then, but fifteen shillings certainly represents low-water mark so far. It cannot be supposed that Professor Wassermann would allow his name to be used unless he was satisfied as to the efficiency of the Institute; and those hospitals which do not pay a whole-time bacteriologist, but send their specimens to private laboratories, will do well to note the rates at which work will be accepted at Sutherland House.

### A NEW SCHOOL FOR EPILEPTIC CHILDREN.

THE Education Committee of the London County Council has approved in principle the provision of a residential school for epileptic children to accommodate a maximum of fifty scholars. This is an interesting decision because of certain conflicting suggestions on which the special schools sub-committee had to report. For instance, co-operation with existing agencies was found virtually impracticable, because of their unwillingness to accept certain cases which had received the recommendation of the Council's medical officer. Again the question of dual control has caused difficulties. The sub-committee therefore came to the conclusion that the only satisfactory alternative was for the Council to establish its own epileptic school. At first, it is suggested, only patients likely to improve under treatment should be accepted. The cost of establishing the school is expected to be £11,800. Monyhull colony is such a shining example of the success which epileptic colonies can achieve that the progress of the proposed school for epileptic children will be watched with interest.

### CHOLERA PATIENTS IN CONSTANTINOPLE.

THE cholera hospital which has been opened at San Stefano, a suburb of Constantinople, is interestingly described by a special correspondent of the *Times*. We quote some of the more technical extracts: "Some of the Turkish soldiers at San Stefano are sick and dying from a disease that in many points resembles cholera, and others are probably dying from cholera. San Stefano is deserted. . . . Then somebody had the idea of making a deserted Greek school into a hospital, and when I first visited this spot I found it under the charge of a Turkish medical officer, assisted by a small group of Europeans. Two ladies and three men of different nationalities! There are in the Greek school eight rooms altogether. There are five rooms occupied by patients, one which forms a sort of kitchen and store room, and two rooms occupied by the medical staff of the Turkish Red Crescent. Besides this there is a compound roofed over in the open air, and there are a certain number of tents. In this house and in these tents there are (or rather there were until to-day) about three hundred and fifty men, all in various stages of sickness. . . When you think of a hospital you think of all the luxury of modern science, of clean beds, a supply of sterilised water, antiseptics, lemonade, baths, etc. Here there are no such appliances. Mattresses on the floor, crowded rooms, no means of washing and dressing or bandaging—a hospital such as the hospitals in the Middle Ages must have been. The Turkish medical authorities have taken steps in the matter of this little hospital. Although it is impossible to persuade any of the owners of the deserted homes at San Stefano to let them be used as hospitals, a house has been found for the medical staff of the English contingent of the Red Crescent. . . The ruling spirit of the Greek school of San Stefano is a lady, who would much dislike her name to be even mentioned. But it has been mentioned, and it may perhaps be permitted to an eye-witness of her untiring and pauseless work and of her angelic goodness to say that Miss Alt came to San Stefano like an angel to hell." The writer forgets that even within living memory institutions "such as hospitals in the Middle Ages must have been" existed a good deal nearer home, but nothing better proves the hospital progress of the past fifty years than his horror of a hospital run on other than the latest scientific principles.

### A DANGEROUS CASINO.

Mentone, to those who know the beauties of the inland scenery in its immediate vicinity, and the attractiveness of the many walks there to be enjoyed, must desire to see evidence of a higher intelligence exhibited by its municipal authorities, who owe the public a debt. On economical grounds and other considerations and for beauty of scenery and climate, Mentone may justly claim to be one of the most attractive places on the Riviera. In recent years a magnificent casino has been erected. How sad is it, therefore, that those responsible for the design, erection, and administration of this casino should suffer a state of affairs which makes it one of the most unpleasant and unhealthy, not to say dangerous, casinos to be met with in the South of France. This undesirable notoriety is due to the insanitary arrangements which prevail in connection with the lavatories and other necessary portions of a great building of this kind. When we visited it on a beautiful day last season the smells which we had to encounter on entering the casino and the condition of the sanitary arrangements filled us with disgust. This state of things will prove dangerous to the public health if it is permitted to continue another season. Last season it reflected the greatest discredit upon everyone connected with the management of the casino, as well as upon the corporation or health authorities, who are responsible for the perfect hygienic conditions of a public building of this kind. We shall be glad to hear that the matter has engaged the attention of those who have it in their power to redress the evils complained of, and we await an assurance that steps have been taken to place the casino in a state of hygienic efficiency.

### A PHARMACIST AND THE PRISON SERVICE.

Mr. Frederic Bullen, M.P.S., has been appointed pharmacist to H.M. Prison, Dartmoor, by the Prison Commissioners, and will take up the duties in January next. Mr. Bullen qualified as a pharmacist in 1905, and has been engaged at the Brixton Dispensary for ten years. He has contributed to these columns and to the pharmaceutical press articles on ancient pharmaceutical history and other subjects. He is a past chairman and secretary of the National Union of Assistant Pharmacists, member of the Council of the Public Pharmacists' Association, and a life member of the Pharmaceutical Society.

### PHARMACISTS AND THE INSURANCE ACT.

The revised regulations as to medical benefit under the Insurance Act, in so far as they relate to the dispensing of medicines and the supply of drugs, differ in minor details only from the provisional regulations issued two months ago. The new provisions recognise more clearly the position of doctors in rural districts in regard to dispensing, but there is no departure from the principle laid down in the Act that, except in special cases, arrangements for dispensing are not to be made with medical practitioners. The question of prices for drugs, which to the pharmacist is naturally an important one, has yet to be considered, as also has another question—namely, What is to happen if the drug bills exceed the amount available for paying them? If, as has been intimated, the chemists' bills are to be discounted if the Drug Fund runs short, the position cannot be regarded as satisfactory, but it is not easy to believe chemists will not be paid in full for goods they actually supply.

### THIS WEEK'S DRUG MARKET.

There has been a slightly improved demand in the Drug market during the week, but until the second or third week in the New Year there is not likely to be any lasting improvement in business. Makers of chloroform have announced a small advance in their prices. Quicksilver has been reduced in price by the importer. Phenacetin is dearer, and a further advance in the quotation for santonin is expected daily. Cream of tartar and tartaric acid are tending slightly lower in value. Oil of sandalwood is again dearer. The Opium market is very dull and the price seems inclined downwards, but it is difficult to form a judgment concerning the immediate future of this drug.

